# ITGB1-mediated molecular landscape and cuproptosis phenotype induced the worse prognosis in diffuse gastric cancer

**DOI:** 10.3389/fonc.2023.1115510

**Published:** 2023-03-16

**Authors:** Xingyu Zhu, Hao Chen, Han Li, Huicheng Ren, Chunshui Ye, Kang Xu, Jin Liu, Fengying Du, Zihao Zhang, Yuan Liu, Xiaozhou Xie, Mingfei Wang, Tianrong Ma, Wei Chong, Liang Shang, Leping Li

**Affiliations:** ^1^ Department of Gastrointestinal Surgery, Shandong Provincial Hospital Affiliated to Shandong First Medical University, Jinan, China; ^2^ Department of Gastrointestinal Surgery, Shandong Provincial Hospital, Shandong University, Jinan, China; ^3^ Key Laboratory of Engineering of Shandong Province, Shandong Provincial Hospital, Jinan, China; ^4^ Medical Science and Technology Innovation Center, Shandong First Medical University & Shandong Academy of Medical Sciences, Jinan, China; ^5^ Clinical Research Center of Shandong University, Clinical Epidemiology Unit, Qilu Hospital of Shandong University, Jinan, Shandong, China; ^6^ Department of Gastroenterological Surgery, The First Affiliated Hospital of Shandong First Medical University, Jinan, China; ^7^ Research Center for Experimental Nuclear Medicine, School of Basic Medical Sciences, Shandong University, Jinan, Shandong, China

**Keywords:** diffuse gastric cancer, Integrin β1, metabolism, cuproptosis, molecular landscape

## Abstract

Diffuse type gastric cancer was identified with relatively worse prognosis than other Lauren’s histological classification. Integrin β1 (ITGB1) was a member of integrin family which played a markedly important role in tumorigenesis and progression. However, the influence of ITGB1 in diffuse gastric cancer (DGC) remains uncertain. Here, we leveraged the transcriptomic and proteomic data to explore the association between ITGB1 expression and clinicopathologic information and biological process in DGC. Cell phenotype experiments combined with quantitative-PCR (q-PCR) and western blotting were utilized to identify the potential molecular mechanism underling ITGB1.Transcriptomics and proteomics both revealed that the higher ITGB1 expression was significantly associated with worse prognosis in DGC, but not in intestinal GC. Genomic analysis indicated that the mutation frequency of significantly mutated genes of ARID1A and COL11A1, and mutational signatures of SBS6 and SBS15 were markedly increased in the ITGB1 low expression subgroup. The enrichment analysis revealed diverse pathways related to dysregulation of ITGB1 in DGC, especially in cell adhesion, proliferation, metabolism reprogramming, and immune regulation alterations. Elevated activities of kinase-ROCK1, PKACA/PRKACA and AKT1 were observed in the ITGB1 high-expression subgroup. The ssGSEA analysis also found that ITGB1 low-expression had a higher cuproptosis score and was negatively correlated with key regulators of cuproptosis, including FDX1, DLAT, and DLST. We further observed that the upregulated expression of mitochondrial tricarboxylic acid (TCA) cycle in the ITGB1 low-expression group. Reduced expression of ITGB1 inhibited the ability of cell proliferation and motility and also potentiated the cell sensitive to copper ionophores *via* western blotting assay. Overall, this study revealed that ITGB1 was a protumorigenic gene and regulated tumor metabolism and cuproptosis in DGC.

## 1 Introduction

Worldwide, the number of deaths due to gastric cancer accounts for 7.7% of the deaths due to tumors every year, ranking fourth, which seriously threatens human health and attracts the attention of numerous medical practitioners (1). There are many histological classification methods for gastric cancer, and some of them are commonly used, including Lauren classification (2, 3). According to Lauren classification, gastric cancer is mainly divided into two types: intestinal-type gastric cancer (IGC) and diffuse-type gastric cancer (DGC), in addition to mixed type and undetermined type (4, 5). IGC and DGC are significantly different in pathogenesis, epidemiology, and clinicopathology perspective, and the pathogenesis of IGC is closely related to environmental factors (6). The pathogeny of DGC appears to be independent of environmental influences, with a relatively young age of onset, multiple in women, and often a familial background (7–9). In relative terms, the incidence of DGC has increased, and some progress has been made in recent years in the study of gastric cancer, but little is known about the molecular landscape of DGC (10). Therefore, a comprehensive understanding of the genetic alterations and expression perturbations underlying DGC heterogeneity is necessary to guide the diagnosis and treatment of DGC.

Integrin β1 (ITGB1) is a member of the integrin family (11–13). The integrin family contains at least 18 α subunits and 8 β subunits, which can form 24 integrins known to have different tissue distribution and overlapping ligands specificity (14, 15). Integrins were α/β heterodimeric cell surface receptors that play a key role in cell adhesion (16) and migration (17, 18) as well as growth and survival (19, 20). The beta-1 subfamily includes 12 unique integrin proteins that bind different extracellular matrix molecules, control extracellular integrin binding, and affect cell adhesion or migration (21, 22). Meanwhile, intracellular signal transduction information transcribed from the cytoplasmic tail domain of beta-1 cells contributes to the regulation of cell proliferation (23, 24), cytoskeletal reorganization, and gene expression (25, 26). Some studies have also demonstrated that ITGB1 was involved in a variety of activities, including embryonic development (27, 28) and blood vessel (29–31), as well as tumor metastasis (32, 33) and angiogenesis (34). There is growing appreciation that ITGB1 was related to the occurrence and development of tumors, such as pancreatic cancer (35, 36), breast cancer (26, 37), and lung cancer (38–40). While the roles of ITGB1 in gastric cancer were remain unknown, especially in DGC.

The mutational signatures were that ongoing mutational processes reflected biological processes active in cancer and could be used as biomarkers to monitor treatment response or as therapeutic anticancertargets (41–43). For example: signature 1 represented the deamination of methylated cytosine which was a process that occurred throughout life, signature 4 could match the signature of smoking and signature 7 was associated with ultraviolet (UV) radiation exposure and also maybe indicate errors in the DNA repair pathway. These mutational signatures caused higher tumor mutational burden and diverse immune response (44). Gains and losses of DNA, also known as copy number variants (CNV), are prevalent in cancer and contribute to cancer initiation, progression and therapeutic resistance (45, 46). However, these genomic alterations on DGC in relation to ITGB1 have not been well described.

Copper is a microscale substance participating in various biological processes. The deregulation of intracellular and extracellular copper homeostasis may influence the biological processes, while recent studies showed that copper-mediated cytotoxicity, named “cuproptosis”, changed the development and progression of cancer. Although cuproptosis occurred by the insufficient or excessive abundance of heavy metal ions, cuproptosis was a novel cell death pathway distinct from ferroptosis, which depended on the mitochondrial tricarboxylic acid cycle rather than glycolysis. Deregulation of copper homeostasis made mitochondrial stress, which gathered lipoylated mitochondrial enzymes and lose related Fe-S cluster proteins, and then triggered cuproptosis (47–49). Some studies have demonstrated that altered Cu homeostasis is directly related to the progression of several neurodegenerative diseases, including Alzheimer’s disease (AD), Huntington’s disease (HD), and amyotrophic lateral sclerosis (ALS) (50). At present, a growing appreciation of the effect of Copper dependent death on tumor progression and prognosis, such as: bladder cancer (51), triple-negative breast cancer (52) and clear cell renal cell carcinoma (53). However, there is a research gap on cuproptosis in DGC.

ITGB1 is one of the most common genes that associated with adhesion in the tumor. However, its potential association with patient’s survival and tumor biology in DGC remains undiscovered. In this study, we found that ITGB1 expression was associated with DGC patient’s prognosis by analyzing transcriptomic and proteomic sequencing dataset. Downregulation of ITGB1 also inhibited the DGC cell proliferation and motility abilities. Through enrichment analysis, we found that ITGB1 was associated with tumor metabolism and immune regulation. We further explored the genomic alteration and identified that the defective DNA mismatch repair mutational signature was associated with ITGB1 expression dysregulation. Given that ITGB1 was significantly correlated with the cuproptosis-related genes, novel strategies triggered to cuproptotic cell death was expected to regulate the fate of ITGB1 overexpressed DGC. Results explored in this study can enhance understanding of molecular mechanism and guide the targeted therapeutic application of ITGB1 for the DGC.

## 2 Materials and methods

### 2.1 Genomic and clinical data

Transcriptomic data were obtained from ACRG, TCGA and CPTAC cohorts and proteomics data were obtained from PKU and CPTAC cohorts. Phosphoproteome were mainly derived from CPTAC cohort. A total of 142 samples from ACRG cohort, 68 samples from TCGA cohort, 74 samples from CPTAC cohort and 82 samples from PKU cohort were included in this study. DGC patients were divided into ITGB1 high expression and low expression subgroup based on the median expression (RNA or protein level) value as cutoff. Somatic mutations (MuTect2), CNV data (SNP array) and clinical data of the selected DGC cohort were extracted from TCGA cohort (https://portal.gdc.cancer.gov). The GISTIC score and gene copy number amplification and deletion data for each sample were analyzed by GISTIC 2.0 software and plotted by maftools package (54, 55). All extracted DNA and RNA for sequencing were obtained from primary untreated tumor tissues. Association of ITGB1 subgroup with clinical information, including age, gender, clinical stage, overall survival, and molecular subtype, was also collected from these studies and is presented in **Table S1**. For proteomics and phosphoproteomics, iTRAQ-labeld LC-MS/MS analysis was performed, and the relative protein abundance of each gene was log2 transformed and centered at zero to obtain the final relative abundance values, which were constructed into an expression matrix. Differential analysis and enrichment analysis were subsequently performed according to different subgroups of ITGB1.

### 2.2 Deciphering the mutational signature operative in the genome

Mutational signatures exacted from aggregated DGC samples (n = 68) genomic data by using the previous framework (56). The method based on variant non-negative matrix factorization can automatically find the appropriate number of mutational signatures. The mutation portrait matrix A was divided into two nonnegative matrices W and H. The matrices W represented mutational processes and the matrices H represented the corresponding mutational activities. The rows of matrix A represents the 96 mutational contexts which rooted in combinations of 6 mutational types (i.e., C > A, C > G, C > T, T > A, T > C, and T > G) and their 5′- and 3′-adjacent bases, and the columns represents the DGC samples. The extracted mutational portrait of DGC was compared and annotated by cosine similarity analysis against the COSMIC (57, 58).

### 2.3 GSEA and network analysis

The R package limma (59) was used to evaluate the differential expressed genes in DGC samples with ITGB1-low and ITGB1-high groups. Briefly, we inputted the normalized expression data into lmFit and eBayes functions, the differential statistics were calculated by the limma package. Afterwards the logFC produced by limma performed GSEA referring to the KEGG reference gene set (download from MSigDB database v7.1). The enrichment plot obtained from the fast gene set enrichment analysis algorithm was implemented in the Bioconductor R package fgsea.

### 2.4 PTM-SEA and KSEA analysis

The variation in biological processes among different ITGB1 expression subgroups were investigated site-centric PTM Signature Enrichment Analysis (PTM-SEA) on phosphoproteomics data sets with the PTM signatures database (PTMsigDB) (60). Kinase-substrate enrichment analysis (KSEA) were performed by KESA App website (https://casecpb.shinyapps.io/ksea/) using phosphosite data according to its manual with the cutoff of p < 0.05 and substrate count more than 1.

### 2.5 Inference of infiltrating cells in the TME

The 64 immune and stromal cell types (spanning multiple adaptive and innate immunity cells, hematopoietic progenitors, epithelial cells, and ECM cells) were inferred by the gene signature–based xCell algorithm (61). Gene expression profiles were prepared using standard annotation files, and data were uploaded to the xCell web portal (https://xcell.ucsf.edu/), with the algorithm run using the xCell signature.

### 2.6 Gastric cancer cell line and drug sensitivity analyses

Available clinical annotation and expression profile of human gastric cancer cell lines (N=40) were obtained from the Broad Institute Cancer Cell Line Encyclopedia (CCLE) project (https://portals.broadinstitute.org/ccle/). Gene dependency screening system (CERES and RNAi) and drug sensitivity database (GDSC1 and PRISM) were accessed from the dependency map (DepMap) portal (https://depmap.org/portal/).

### 2.7 Cell culture

MKN-45 cells were kindly provided by Key Laboratory for Experimental Teratology of the Ministry of Education, Department of Pathology, School of Basic Medical Sciences, Shandong University. MKN-45 cells were maintained in 1640 medium (Gibco) supplemented with fetal bovine serum (FBS, 10% PAN), penicillin (100U/mL, Thermo Fisher), and streptomycin (100U/mL, Thermo Fisher) and cultured in 95% air and 5% CO_2_ at 37°C. The 1% O_2_ stimulation was maintaining MKN-45 cells by 1640 medium (Gibco) supplemented with fetal bovine serum (FBS, 10% PAN), penicillin (100U/mL, Thermo Fisher), and streptomycin (100U/mL, Thermo Fisher) and cultured in 94% N_2_, 5% CO_2,_ and 1% O_2_ at 37°C.

### 2.8 Cell transfection

Lentivirus particles of shNC (Negative control), shITGB1-1, shITGB1-2 (ITGB1 knockdown), Vector (vector control), or ITGB1 (ITGB1 overexpression) for humans were purchased from Genomeditech. MKN-45 cells were infected by 1640 medium (Gibco) supplemented with fetal bovine serum (FBS, 10% PAN), penicillin (100U/mL, Thermo Fisher), lentivirus, polybrene (0.1%) and streptomycin (100U/mL, Thermo Fisher) for 48h and selected with puromycin (0.5μg/ml, MedChemExpress) for 7 days.

### 2.9 Quantitative real-time PCR

Total RNA from cells was extracted by Trizol reagent (Vazyme, China). RNA was reversely transcribed into cDNA by HiScript III RT SuperMix for qPCR (Vazyme, China) following the manufacturer’s instructions. The quantitative real-time polymerase chain reaction (qRT-PCR) method was used to detect mRNA expression levels by the ChamQ Universal SYBR qPCR Master Mix (Vazyme, China) protocol on Applied Biosystems QuantStudio 1 Real Time PCR system (Applied Biosystems, ThermoFish). The primer sequences were used for qRT-PCR as follows: for ITGB1, 5′- GTCGTGTGTGTGAGTGCAAC-3′ (forward), 5′- GCTGGGGTAATTTGTCCCGA′ (reverse). GAPDH was used as reference for mRNA. The relative expression levels of mRNA were calculated by using the 2−ΔΔCt method in which higher 2−ΔΔCt reflects higher expression.

### 2.10 Western blotting analysis and antibodies

In brief, total cell lysates were prepared with cell lysis buffer. After denaturing *via* boiling, total protein was quantified using a BCA protein assay kit (Solarbio). Equivalent amounts of protein were separated by SDS-PAGE at 80 V for 2.5h and transfected to PVDF membranes for 1.5h. The membranes were washed using 1% TBST by three cycles of 5 minute after incubation with Primary antibodies targeting ITGB1 (Proteintech, 12594-1-AP), FDX1 (Proteintech, 12592-1-AP), LIAS (Proteintech, 11577-1-AP), DLD (Proteintech, 16431-1-AP), DLST (abcam, ab177934), DLAT (Proteintech, 13426-1-AP), PDHA1 (Proteintech, 18068-1-AP), PDHB (Proteintech, 14744-1-AP), GLS (Proteintech, 29519-1-AP) and β-actin (Proteintech, 20536-1-AP) at 4°C overnight. Then membranes were treated with secondary antibodies (Proteintech, SA00001-2).

### 2.11 CCK-8 assay

Cell proliferation assays were performed with Cell Counting Kit-8 (DojinDo, Japan) according to the manufacturer’s protocol and detected at 0h, 24h, 48h, 72h and 96 h.

### 2.12 Colony formation

Knockdown or forced overexpression of ITGB1 in MKN-45 cells (2×103cells/well) were seeded into 6-well plates and cultured for 1 weeks at 37°C, and the culture medium was replaced with fresh medium every 4 days. Then, cells were washed using PBS, fixed with 4% paraformaldehyde for 30 min at room temperature, and stained with 0.5% crystal violet for 30 min at room temperature. The number of colonies (containing >50 cells) was observed and counted using an optical microscope.

### 2.13 Migration and invasion assay

For migration and invasion analysis, cell (migration: 3 × 105/mL, invasion: 5 × 105/mL) suspension (200 μl of serum-free medium) were seeded onto 8-mm Pore Transwell Inserts (Corning) coated with Matrigel for invasion assay, or without Matrigel for migration assay. Lower chambers were filled with complete medium (600 μl). Cells on the Transwell Inserts were then fixed with paraformaldehyde/PBS (4%) for 30 min. Next, fixed cells were stained with hematoxylin solution (Sigma-Aldrich) for 30min. Then microphotograms of the cells migrated onto the lower side of the filter were imaged using a microscope. From the microphotograms, cells that migrated or invaded onto the lower side of the filter were manually counted. Cell numbers were quantified from ten randomly selected fields with the same area.

### 2.14 Wound healing assay

Knockdown or forced overexpression of ITGB1 in MKN-45 cells were seeded into 6-well plates and allowed to grow until > 95% confluence. And then the cell layer was gently scratched through the central axis using a sterile plastic tip and loose cells were washed away by PBS and the media replaced with serum free media. Quantification of cell motility by measuring the distance between the invading fronts of cells in three random selected microscopic fields (×100) for each condition and time point (0h, 24h, 48h).

### 2.15 Statistical analysis

Statistical analyses in this study were generated by R-4.0.1. For quantitative data, statistical significance for normally distributed variables was estimated by Student’s 2-tailed t-tests, and non-normally distributed variables were analyzed by the Wilcoxon’s rank-sum test. The χ2 test and Fisher’s exact test were used to analyze contingency tables depending on specific grouping condition. Correlations between two quantitative variables were then analyzed and described using Spearman’s correlation coefficient. Kaplan Meier survival analysis were used to analyze the survival with R survival package (survminer 2.40-1). Cox regression analysis was used to test the association between ITGB1 and survival outcomes, controlling for age, gender, stage. Experiment statistical analyses were carried out using Prism 8 (GraphPad). Data were obtained from at least three independent experiments and for statistical significance were analyzed using Student’s t-test or Wilcoxon’s rank-sum test. P < 0.05 was considered statistically significant.

## 3 Results

### 3.1 ITGB1 predictive of survival in DGC

**Figure 1:**
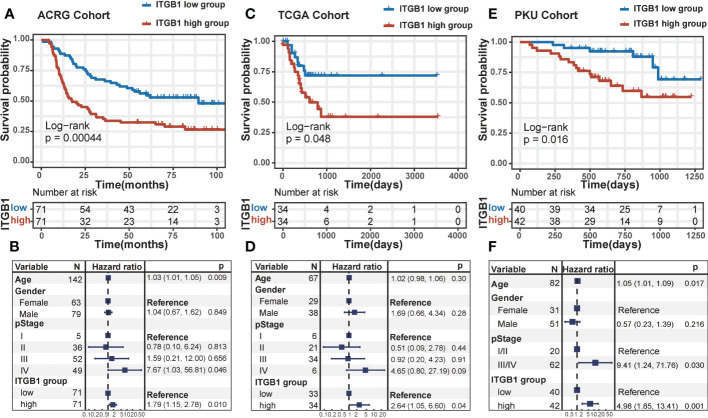
The survival analysis of ITGB1 in human DGC Kaplan-Meier curves of relapse-free survival according to ITGB1 high and low groups in the ACRG diffuse-type GC cohort **(A)** TCGA diffuse-type GC cohort **(C)** and PKU diffuse-type GC cohort **(E)**. Forest plot representation of the Multivariate Cox regression model delineated the association between ITGB1 and survival in the three cohorts **(B, D, F)**. Age, gender or stage were taken into account.

DGC patients were divided into ITGB1 high and low expression subgroup based on the median expression (RNA or protein level) value as cutoff. In Kaplan-Meier survival analysis, we found that the ITGB1-low subgroup has a better survival outcomes compared with the ITGB1-high subgroup among the DGC from transcriptomic dataset (ACRG cohort: P = 0.00044, **Figure 1A**; TCGA cohort: P = 0.048, **Figure 1C**; log-rank test) and proteomic dataset (PKU cohort: P = 0.016, **Figure 1E**; log-rank test). When controlled for age, gender, and stage in multivariate Cox proportional hazards regression analysis, we found that the ITGB1 remains significantly associated with better prognosis in the three cohorts (ACRG cohort: HR, 1.79 [95%CI, 1.15–2.78], P = 0.010, **Figure 1B**; TCGA cohort: HR, 2.64 [95%CI, 1.05–6.60], P = 0.04. **Figure 1D**; PKU cohort: HR, 4.98 [95%CI, 1.85–13.41], P = 0.001, **Figure 1F**). However, the prognosis between the two ITGB1 subgroups was not significant in the intestinal-type gastric cancer of ACRG and TCGA cohort (ACRG cohort: P = 0.5, **Figure S1A**; TCGA cohort: P = 0.18, **Figure S1B**; log-rank test, IGC samples was not existed in PKU cohort). In short, ITGB1 could be a potential prognostic indicator for patients with DGC,

### 3.2 ITGB1 as a protumorigenic factor in DGC

**Figure 2:**
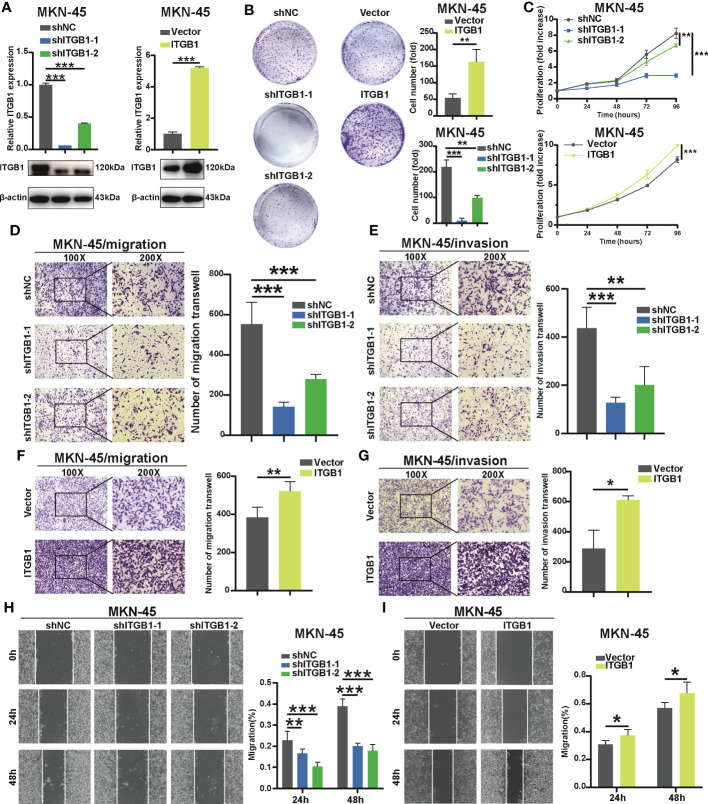
Effect of ITGB1 knockdown or forced ITGB1 expression in DGC cells **(A)** Confirmation of knockdown or forced expression of ITGB1 in MKN-45 cells by Quantitative-PCR (q-PCR) combined with Western blotting analysis. Cell proliferation assay in MKN-45 cells with shNC (negative control), shITGB1-1, shITGB1-2 (ITGB1 knockdown), Vector (vector control), or ITGB1 (ITGB1 overexpression) by colony formation **(B)** and cell counting kit-8 (CCK8) assays **(C)**. Cell migration **(D)** and invasion **(E)** assays in MKN-45 cells with shNC (negative control), shITGB1-1, shITGB1-2 (ITGB1 knockdown). Cell migration **(F)** and invasion **(G)** assays in MKN-45 cells with Vector (vector control), or ITGB1 (ITGB1 overexpression). **(H)** and **(I)** Cell migartion assay in MKN-45 cells with shNC (negative control), shITGB1-1, shITGB1-2 (ITGB1 knockdown), Vector (vector control), or ITGB1 (ITGB1 overexpression). *, P<0.05; **, P<0.01; ***, P<0.001.

To determine the previous findings on the association of ITGB1 with DGC, we further explored the biological mechanism from a cellular perspective. We generated MKN-45 cells with ITGB1 knockdown and overexpression by shRNA (sh-ITGB1) (**Figure 2A**). According to colony formation assay (**Figure 2B**) and cell counting kit 8 (CCK8) assay (**Figure 2C**), knockdown of ITGB1 in MKN-45 cells markedly decreased cell proliferation. While overexpression of ITGB1 markedly increased cell proliferation. In addition, transwell assay (**Figures 2D–G**) and wound healing assay (**Figures 2H, I**) showed that ITGB1 knockdown also significantly decreased the migration and invasion. Overexpression of ITGB1 markedly increased the migration and invasion. These results suggested that ITGB1 played a protumorigenic role in DGC.

### 3.3 Tumor genomic characteristics in DGC

**Figure 3:**
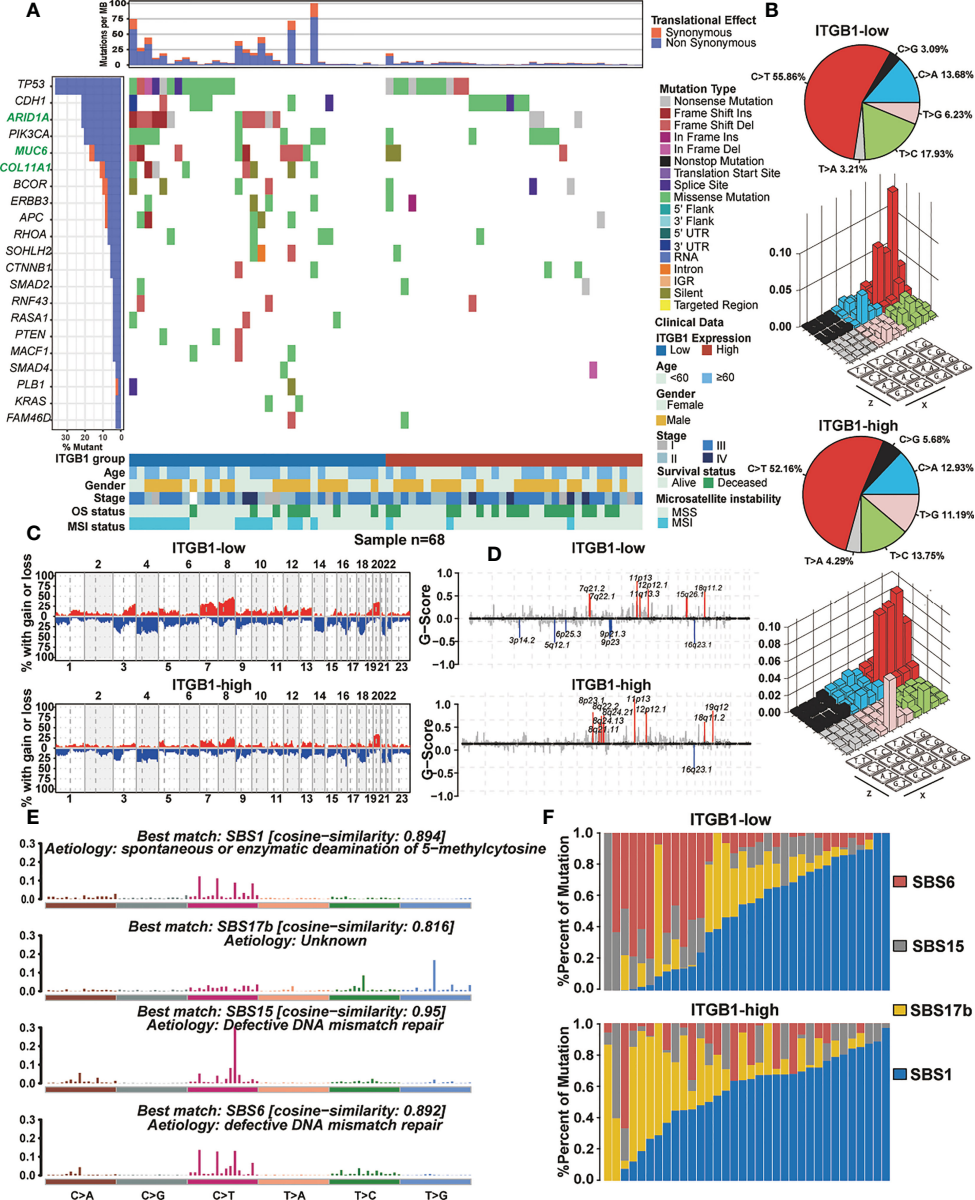
The mutational signature and the chromosomal copy number variation in DGC **(A)** Mutational landscape of significantly mutated genes in TCGA cohort were stratified by the ITGB1-low and the ITGB1-high groups. The middle panel describes the mutation relation of significantly mutated genes across analyzed cases with mutation types color-coded differently. **(B)** Lego plot representation of mutation patterns in the ITGB1-low group and the ITGB1-high group. Single-nucleotide substitutions were divided into six categories with 16 surrounding flanking bases. The pie chart in upper left showed the proportion of six sorts of mutation patterns. **(C, D)** The chromosomal copy number variation of somatic cell in the ITGB1-low and the ITGB1-high groups. The chromosome names were on the x-axes, whereas y-axes was the gain or loss of chromosome copy number mutation frequency in different ITGB1 groups. **(E)** The mutational activities of corresponding extracted mutational signatures (SBS1, SBS17b, SBS15 and SBS6). The trinucleotide base mutation types were on the x axes, whereas y axes showed the percentage of mutations in the signature attributed to each mutation type. **(F)** Mutational exposures (number of mutations) were attributed to each mutation signature. The percentage of mutations were on the y axes.

Somatic mutational profiles of DGC patients from the ITGB1-low group and the ITGB1-high group studies were analyzed in TCGA cohort. We found that the ITGB1-low group had significantly higher tumor non-silent mutation load, compared with the ITGB1-high group (Wilcoxon rank-sum test, P = 0.0089, **Figure S2A**). We next evaluated mutations of individual genes (such as common oncogenic driver mutations, TP53, KRAS, and PTEN; EMT associated genes, CDH1, and CTNNB1) that may be associated with ITGB1 (**Figure 3A**). Samples with ARID1A, MUC6, and COL11A1 mutations were significantly more frequent in ITGB1 low subgroup than high subgroup (Fisher exact test, P < 0.05). Interestingly, mutations in COL11A1, RASA1, PTEN, MCF1, PLB1, and KRAS were only found in ITGB1-low group. Calculating the number of single nucleotide variants in the matrix of 96 possible mutations with trinucleotide background found that predominant mutations in DGC were featured by the C>T transitions at ApCpN trinucleotide sites. Specifically, the C>T transition at ApCpA were highlighted in low ITGB1 subgroup, whereas the T>G transition at GpTpC were elevated in high ITGB1 subgroup (**Figure 3B**), suggested the specific mutational processes operative in ITGB1 subgroup heterogeneity. Subsequently, we analyzed the gene copy number variation of DGC in different ITGB1 expression subgroup. In general, the chromosomal copy number variation (both gain and loss) of the ITGB1-low group was relatively higher than the ITGB1-high group (**Figure 3C**). Focal level SCNAs revealed that the specific cytobands (FDR<0.01) in each ITGB1 subgroup. As shown by genome plot, the cytobands in 7q21.2, 15q26.1 in low ITGB1 score subgroup, and 8p23.1, 8q21.11, 8q24.13, 8q24.21, 19q12 in high ITGB1 subgroup contained the markedly amplified focal regions; cytobands in 3p14.2, 5q12.1, 6p25.3, 9q21.3, 9p23 in low ITGB1 subgroup contained the frequently deleted regions (**Figures 3D**, **S2B**). To further explore the mutational processes operative in patients with DGC, we extracted the mutation signatures from the mutational profile (**Figure S2C**). The identified four mutational signatures (eg. signatures 1, 2, 3, 4) were re-annotated them against the Catalogue of Somatic Mutations in Cancer (COSMIC-v3) signature nomenclature by using cosine similarity analysis (**Figures S2D**, **3E**). We observed that the SBS1 signature consisted largely of C>T transition that was associated with spontaneous or enzymatic deamination of 5-methylcytosine in most cancers. The SBS17b signature was associated with T>G for some unknown reasons. The SBS15 and SBS6 signature was associated with C>T transition due to defective DNA mismatch repair. Meanwhile, we observed that the percentage of mutations of SBS6 signature and SBS15 signature were markedly higher in the ITGB1-low group, while the percentage of mutations of SBS17b was markedly higher in the ITGB1-high group (**Figure 3F**).

### 3.4 Identification of proteins and phosphorylation pathways associated with ITGB1

**Figure 4:**
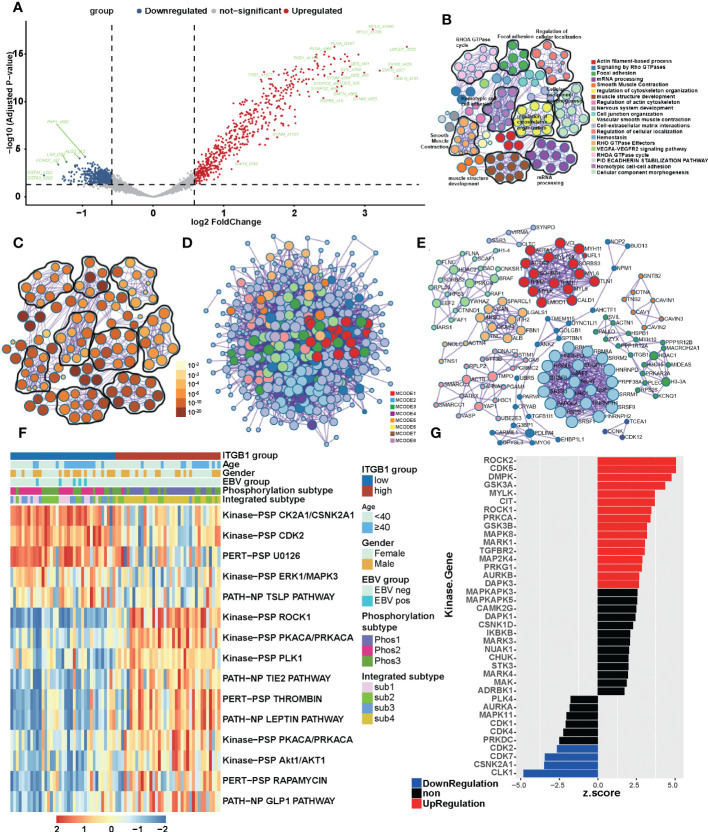
The landscape of biological processes of ITGB1 in DGC by the joint analysis of CPTAC and PKU in the phosphorylation level **(A)** Phosphorylation site regulated by ITGB1 in CPTAC database. Metascape enrichment network visualization summarized different biological processes **(B)** and relevance **(C)** in the phosphorylation level. The name of biological processes were showed in the right of chart. **(D)** Divided the set of proteins that physically interacted into 10 sub-clusters based on the MCODE method and proteins with the same clusters were characterized by the same GO terms and KEGG pathways. **(E)** The landscaoe of protein-protein interacting between and within the MCODE clusters. **(F)** The phosphorylation pathways in different ITGB1 groups were evaluated by the single sample GSEA (ssGSEA). **(G)** Kinase-Substrate Enrichment Analysis (KSEA) revealed the kinases associated with ITGB1.

To get closer to the most primitive manifestation of the DGC, we analyzed it from the phosphoproteome level. It was well known that phosphorylation was one of the most common and important modification patterns of proteins. In CPTAC database we performed a differential analysis and identify the phosphorylation sites associated with ITGB1 (such as: upregulated: TNS1_s1477, TNS1_s1164, FLNA_s966 and FLNA_t2167; downregulated: GSTA1_s202, GSTA3_s202 and KCNQ1_s27) in DGC (**Figure 4A**, **Table S2**). By analysis of ITGB1 combining proteomics and phosphorylation proteomics in the CPTAC and PKU databases (CPTAC: P < 0.01; PKU: P < 0.05), we functional annotated the biological processes associated with ITGB1. In order to further explore the biological significance of ITGB1, we conducted GO enrichment and Metascape analysis in the protein level and phosphorylated protein level. Enriched biological processes summarized that in the phosphorylated protein level, the ITGB1 was concentrated on Actin filament-based process, Signaling by Rho GTPases, Focal adhesion, mRNA processing, and so on (**Figures 4B–D**), and in the protein level, the ITGB1 was characterized by Metabolism of RNA, Translation, Vesicle-mediated transport, Hemostasis, and others (**Figures S3A–C**). Biological processes associated with metabolism raking in the top 50 were Metabolism of RNA, Peptide metabolic process, Cellular amide metabolic process, Selenoamino acid metabolism, and ncRNA metabolic process. By Metascape analysis, we performed protein-protein interaction enrichment analysis (phosphorylated protein level: **Figures 4D, E**; protein level: **Figures S3D, E**). The network generated by enrichment analysis consisted of a series of protein clusters. Therefore, we divided the set of proteins that physically interacted into 10 sub-clusters based on the MCODE method, and proteins with the same clusters were characterized by the same GO terms and KEGG pathways. Cluster MCODE1 associated with Smooth Muscle Contraction (R-HSA-445355), Structural molecule activity (GO:0005198), Muscle contraction (R-HSA-397014), et.al, which consisted of SRPRA, ITGA1, ALB, et al. Cluster MCODE2 associated with Translation (R-HSA-72766), ECM-receptor interaction (HSA04512), which consisted of SPCS2, EIF3E, DDOST, et al. Cluster MCODE3 associated with Regulation of expression of SLITs and ROBOs (R-HSA-901055), Signaling by ROBO receptors (R-HSA-376176), Cellular responses to stress (R-HSA-2262752), et.al, which consisted of PSMD2, STT3B et al. Then according to the enrichment analysis, we drew a heat map of ITGB1 related biological processes (**Figure S3F**) and genes which targeted by and regulated ITGB1 (**Figures S3G, H**). We found lots of biological processes, target genes, and regulated genes associated with ITGB1 (such as target genes: DLX6, FOXG1, HES4, et.al; regulated genes: IRF9, HIF1A, ERG, et.al).

Meanwhile, in order to find out the association of ITGB1 with phosphorylation-related pathways, we applied the differential phosphorylated protein to perform ssGSEA/post-transcriptional modification (PTM) analysis and composed a heatmap to visualize the relative abundance. We found that the phosphorylation processes of kinase CK2A1/CSNK2A1, CDK2, and U0126 (MEK inhibitor) were upregulated in the ITGB1-low group, and ROCK1, PKCA/PRKCA, PLK1, and Lepin were upregulated in the ITGB1-high group (**Figure 4F**). Kinase-Substrate Enrichment Analysis (KSEA) revealed that some kinases (like ROCK2, CDK5, DMPK, GSK3A, MYLK, CIT, ROCK1, PRKCA, GSK3B, MAPK8, MAPK1, TGFBR2, MAP2K4, PRKG1, AURKB, and DAPK3) expression up-regulated and some kinases (like CLK1 CSNK2A1, CDK7, and CDK2) expression down-regulated (**Figure 4G**, **Table S3**). These results further revealed the protein phosphorylation profile underlying ITGB1 dysregulation and provided the comprehensive insights on ITGB1-mediated transcriptional modification.

### 3.5 Molecular features and extracted related pathways associated with ITGB1

**Figure 5:**
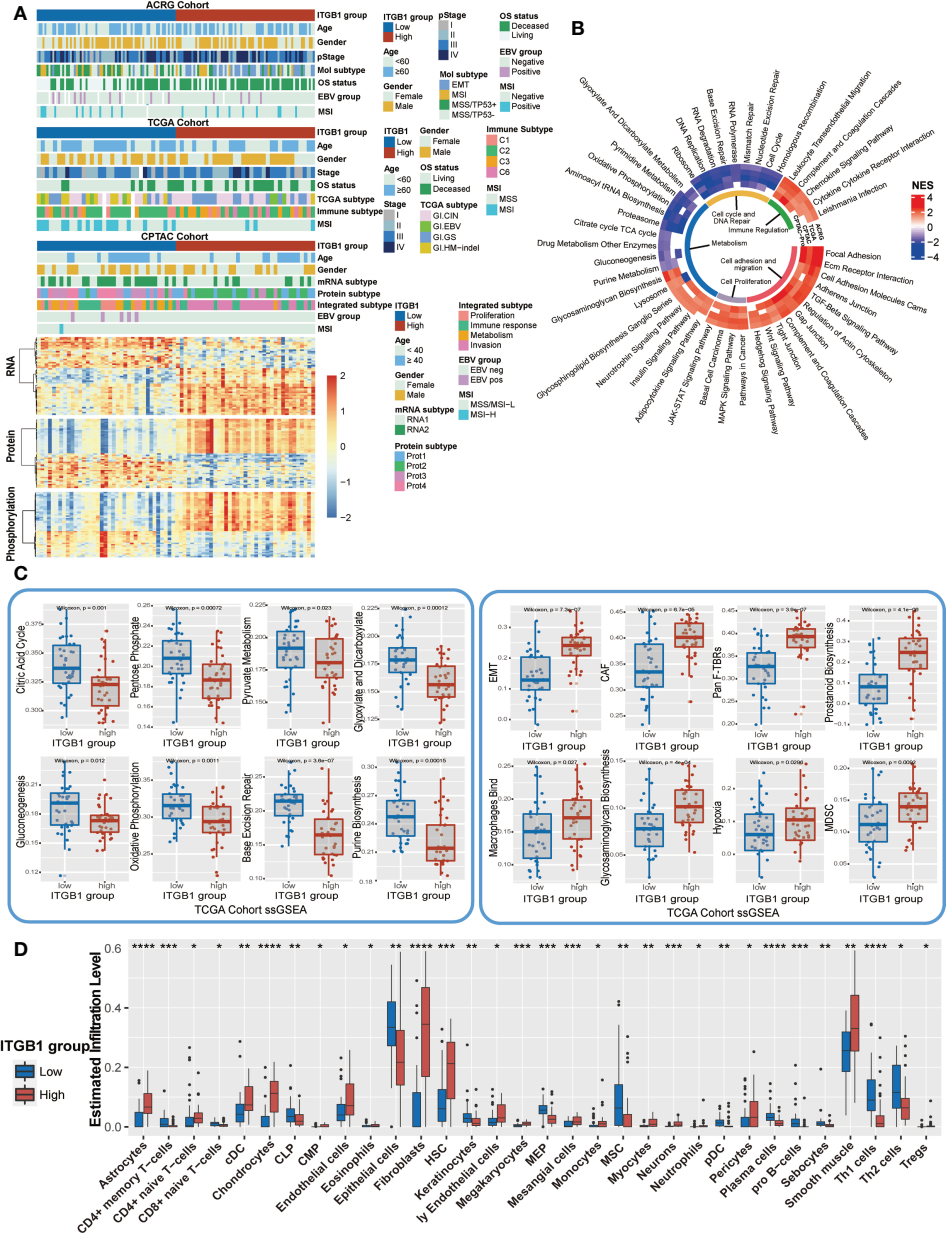
Enrichment analysis of ITGB1 related genes, pathways and immune regulation alteration **(A)** The relative RNA and protein expression of cell proliferation, cell cycle, metabolism, cell adhesion and immune regulation in different ITGB1 groups were evaluated in ACRG TCGA and CPTAC cohorts. **(B)** Enriched analysis in different pathways (Immune regulation, Cell cycle and DNA repair, Metabolism, Cell proliferation and Cell adhesion and migration). **(C)** Related metabolism and other pathways ssGSEA was calculated and compared in different ITGB1 groups from TCGA cohort. **(D)** The relative abundance of tumor-infiltrating leukocytes (TILs) with ITGB1 grouping in diffuse GC from TCGA datasets was estimated by the CIBERSORT algorithm. *, P<0.05; **, P<0.01; ***, P<0.001; ****, P<0.0001.

Previous studies have identified different molecular subtypes nomenclature of DGCs on the basis of transcriptomic and genomic analysis. Here, we also investigated the association of ITGB1 expression subgroup with previous identified clinical and molecular characteristics (**Figures 5A**, **S4A**). Interestingly, the MSS/TP53+ subtype (ACRG-defined), GI.HM-indel and Immune-C2 (TCGA-defined), RNA2 and metabolism subtype (CPTAC-defined), PX1-cell cycle (PKU-defined) were predominantly enriched in ITGB1 low expression subgroup, whereas EMT subtype (ACRG-defined), GI.GS subtype and Immune-C3 (TCGA-defined), RNA1 and invasion subtype (CPTAC-defined), PX2-EMT subtype (PKU-defined) was strongly enriched in ITGB1 high expression subgroup (**Figure 5A** upper panel, **Figure S4A**). The differentially expressed RNA in CPTAC, ACRG, and TCGA cohorts were also illustrated (**Figure 5A** lower panel and **Figure 4B**). The mRNA levels (such as, RAB31, NRP2, ANTXR1, and CLIC4) were upregulated and the mRNA levels (such as, PGC, TFF2, and GKN1) were downregulated in CPTAC, ACRG, and TCGA cohorts (**Table S4**). To explore the effects of ITGB1 on biological process, we performed GSEA analysis with KEGG database on RNA levels from ACRG, TCGA, and CPTAC cohorts; and protein levels from CPTAC cohorts. GSEA with ring heatmap indicated that ITGB1 high expression was significantly enriched in immune inflammation, cell adhesion and migration, tumorigenesis pathway, whereas the ITGB1 low expression was predominately enriched in cell cycle and DNA repair, metabolism (**Figure 5B**). Interestingly, metabolism pathway related to aerobic respiration (citrate cycle TCA cycle, oxidative phosphorylation, drug metabolism other enzymes) and glycan-related circuits were negatively associated with ITGB1 expression. We further performed the ssGSEA analysis by using the identified immune-oncology signatures curated form Zeng et al. studies (62). ITGB1-low group had a significantly higher enrichment score compared with the ITGB1-high group in metabolism (citric acid cycle, pentose phosphate, pyruvate metabolism, glyoxylate and dicarboxylate metabolism, gluconeogenesis, oxidative phosphorylation, and purine biosynthesis) and DNA Repair (Base Excision Repair). In contrast, the cell migration and cell matrix (EMT, CAF, Pan F-TBRs) and tumor inflammation (Macrophages Bind, Hypoxia, MDSC) had significantly more enrichment in the ITGB1-high group (Wilcoxon rank-sum test, **Figure 5C**). These findings were also verified in the ACRG cohort (Wilcoxon rank-sum test, **Figure S4C**).

Furthermore, we evaluated (with the xCell algorithm) the abundance of cell subpopulations in the DGC microenvironment using gene expression data. We found that tumor inflammation cells, fibroblasts cells, and endothelial cells (Astrocytes, cDC, Chondrocytes, Fibroblasts, Hematopoietic stem cells (HSC), Megakaryocytes, Mesangial cells, Monocytes, Neurons, Tregs, Smooth muscle) had a more enrichment in the ITGB1-high group. While CD4+ memory T-cells, common lymphoid progenitors (CLP), Epithelial cells, Keratinocytes, Megakaryocyte–erythroid progenitors (MEP), MSC, plasmacytoid dendritic cells (pDC), Plasma cells, pro B-cells, Sebocytes, and Th1 cells had a better enrichment in the ITGB1-low group (**Figure 5D**).

### 3.6 ITGB1 mediated cuproptosis

**Figure 6:**
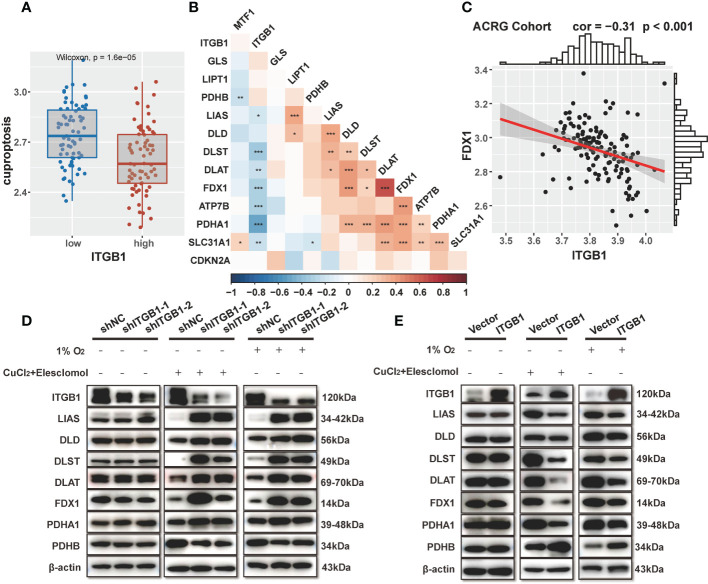
Association of ITGB1 with cuproptosis in DGC **(A)** Cuproptosis was stratified by ITGB1. **(B)** Correlations between ITGB1 and the cuproptosis-related-gene using Spearman analysis. The negative correlation was marked with blue and positive correlation with red. **(C)** Correlation analysis between ITGB1 and FDX1 in ACRG cohort. **(D)** Western blotting analysis of ITGB1, LIAS, DLD, DLST, DLAT, FDX1, PDHA1, PDHB and β-actin in MKN-45 cells with or without knockdown. **(E)** Western blotting analysis of ITGB1, LIAS, DLD, DLST, DLAT, FDX1, PDHA1, PDHB and β-actin in MKN-45 cells with or without overexpression. *, P<0.05; **, P<0.01; ***, P<0.001.

Previous enrichment analysis revealed that ITGB1 was negatively associated with mitochondria TCA metabolism. As one of the metabolism of the most important substance, glucose metabolism is vital for organisms to maintain homeostasis. Recent studies showed that cuproptosis, a novel form of cell death, was based on glucose metabolism, which was characterized by mitochondrial tricarboxylic acid (TCA) cycle and protein lipoylation (47, 48). In consideration of previous findings that the citric acid cycle was higher in the ITGB1-low group, we further explored the association of ITGB1 and cuproptosis signature. Further analysis indicated that cuproptosis signature score was markedly higher in the ITGB1-low group rather than in the ITGB1-high group (Wilcoxon rank-sum test, p = 1.6e-5, **Figure 6A**). In Kaplan-Meier survival analysis, we found that the cuproptosis-high group demonstrated a better survival compared with the cuproptosis-low group among the patients with DGC (TCGA cohort: P = 0.036, **Figure S5A**; ACRG cohort: P = 0.048, **Figure S5B**; log-rank test). We examined the relationship between known cuproptosis-related-genes and ITGB1 through Spearman analysis. A heatmap of the correlation matrix demonstrated that ITGB1 was negatively correlated with DLST, DLAT, FDX1, ATP7B, and PDHA1, but positively correlated with PDHB and GLS (**Figure 6B**, **Table S5**). Correlation between FDX1 and ITGB1 was shown in dot plot among ACRG, TCGA and CPTAC cohorts (ACRG cohort: Spearman r = -0.31, P < 0.001, **Figure 6C**; TCGA cohort: Spearman r = -0.36, P < 0.001, **Figure S5C**; CPTAC cohort: Spearman r = -0.39, P < 0.001; **Figure S5D**).

Subsequently, we verified the findings *via* molecular experiments. Western blotting analysis indicated that the knockdown of ITGB1 in MKN-45 cells increased the protein levels of LIAS, DLD, DLST, DLAT, FDX1, PDHA1 and reduced the protein level of PDHB by stimulating 1% O_2_ for 8h and 1uM CuCl_2_, 200 nM Elesclomol for 7h (**Figure 6D**). On the contrary, forced overexpression of ITGB1 decreased the protein levels of these genes and increased the protein level of PDHB (**Figure 6E**). These findings suggested that ITGB1 may be involved in cuproptosis in DGC.

### 3.7 ITGB1-associated potential therapeutic compounds

We further investigated the cell viability and drug sensitivity in relation to ITGB1 expression with GC cell model. The cancer-dependent score was analyzed using genetic dependency of RNAi and CRISPR screening dataset from DepMap database (https://depmap.org/portal/download/). Median CERES score in these cell lines was -0.27 (CERES below 0.2 means the gene is an essential gene), and most of the RNAi score were below 0 (RNAi approach to 0 means the gene is not an essential gene, **Figure S6**). These findings indicated that ITGB1 could be regarded as an essential gene in GC.

**Figure 7:**
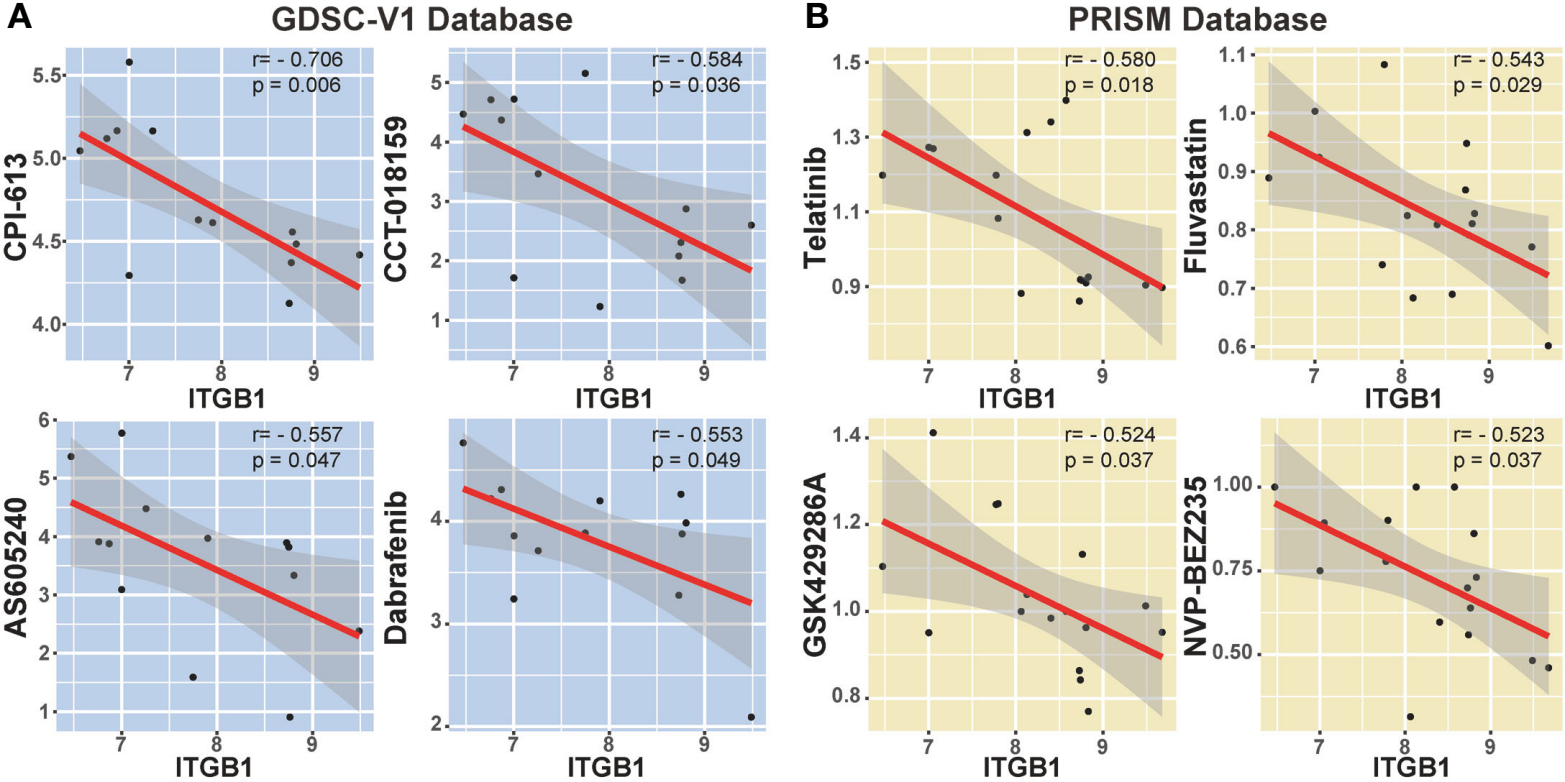
Correlation analysis between ITGB1 and candidate targeted drugs in GDSC-V1 database **(A)** and PRISM V2 database **(B)**.

We also explored the potential compounds in treating the ITGB1 high expressed DGC tumors by using the Genomics of Drug Sensitivity in Cancer (GDSC) database (**Figure 7A**) and Profiling Relative Inhibition Simultaneously in Mixtures (PRISM) database (**Figure 7B**). Agents with significant negative correlation between ITGB1 and drugs IC50 were screened across the GC cell lines (Devimistat: Spearman r = -0.706, P = 0.006; CCT.018159: Spearman r = -0.584, P = 0.036; AS605240: Spearman r = -0.557, P = 0.047; Dabrafenib: Spearman r = -0.553, P = 0.049; Telatinib: Spearman r = -0.580, P = 0.018; Fluvastatin: Spearman r = -0.543, P = 0.029; GSK429286A: Spearman r = -0.524, P = 0.037; NVP-BEZ235: Spearman r = -0.523, P = 0.037; **Figure 7B**, **Table S6**). These potential therapeutic agents were highly negatively correlated with the ITGB1 and may have potential therapeutic implications for patients with high ITGB1.

## 4 Discussion

**Figure 8:**
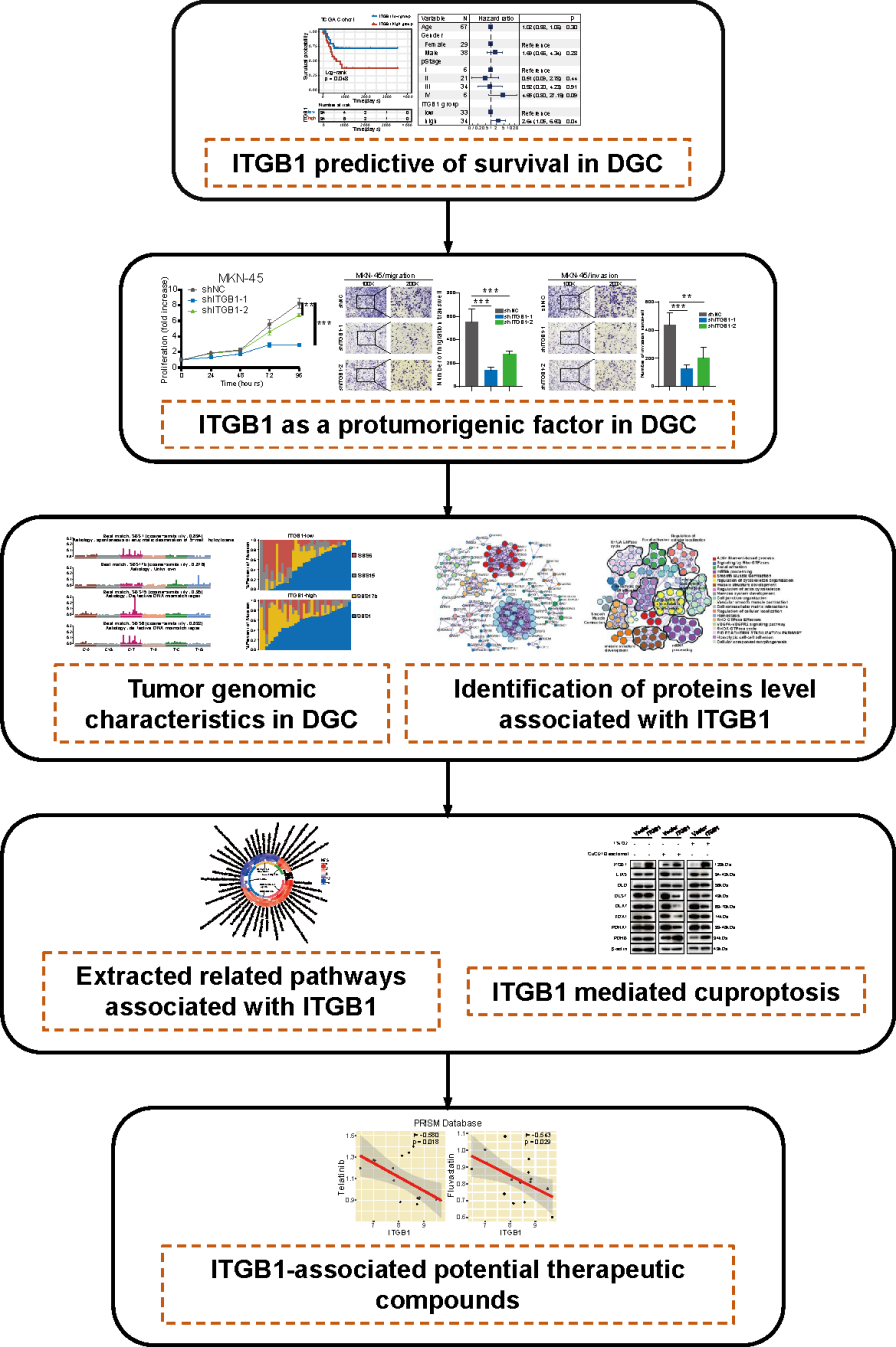
The workflow of tumor mutational burden and metabolic characteristics associated with ITGB1 in DGC.

In this study, we comprehensively analyzed the molecular landscape and clinical relevance of ITGB1 dysregulation in DGC, and revealed the ITGB1 mediated cuproptosis signaling in regulating the tumorigenesis of DGC (**Figure 8**). Cell phenotype experiment demonstrated that ITGB1 was a protumorigenic factor and inducing the proliferation, migration, and invasion properties of DGC. Meanwhile, we extracted tumor genomic characteristics from DGC, and found that ITGB1 was associated with tumor mutation load, dMMR signature, and copy number variations. We further performed a phosphoproteomic analysis to determine the altered pathway in phosphorylation level. By enrichment analysis of ITGB1 differentially expressed molecules, we found that in addition to being associated with tumor adhesion, ITGB1 was also significantly associated with tumor immune and metabolism. Given the significant correlation between ITGB1 and cuproptosis score, further western blotting analysis verified that ITGB1 influenced the cuproptosis-related-genes (such as FDX1, LIAS, DLD, DLST, DLAT, PDHA1, and PDHB). Finally, we analyzed the cell dependency score of ITGB1 in DGC cell lines and analyzed the GDSC and PRISM databases to identify candidate drugs, identifying eight drugs (Devimistat, CCT.018159, AS605240, Dabrafenib, Telatinib, Fluvastatin, GSK429286A, and NVP-BEZ235) that were significantly associated with ITGB1.

In recent studies, ITGB1 was not only able to promote tumor progression by participating in multiple tumor-related signaling pathways such as p53 (63), EMT (34), and PI3K/AKT (20, 64), which regulate the expression of proto-oncogenes or suppressors but also serve as important biomarkers to assess the prognosis of cancer patients (33, 65). Researchers found that ITGB1 was able to influence cell function and thus influence tumor development and progression. For example, in cancer cells, ITGB1 can bind to EpCAM and regulate cell adhesion (66); The high expression of ITGB1 may be related to the poor prognosis of colorectal cancer and can lead to the migration and invasion of colorectal cancer cells (67). CNV caused amplification on oncogenes and the deletion on tumor suppressor genes led to or promoted the occurrence and development of tumors. Previous studies reported that MYC which located on chr8p24.21 locus (amplified in ITGB1 high subgroup), can promote malignant progression of gastric cancer cells (68). GATA6 which located on chr18q11.2 locus (amplified in ITGB1 low subgroup), suppressed migration and metastasis by regulating the miR-520b/CREB1 axis in gastric cancer (69). Fibroblast growth factor 19 (FGF19) which located on chr11q13.3 locus, facilitated the self-renewal of liver cancer stem cells (70). Meanwhile, these genes of phosphorylation related with ITGB1 also facilitated or inhibited the occurrence and development of tumor. ROCK2 in gastric cancer cell promoted tumor cell proliferation, metastasis and invasion (71). CDK5 suppressed the metastasis of gastric cancer cells (72). MYLK repressed gastric cancer progression (73). The overexpression of ROCK1 can promote proliferation (74), invasion and migration (75, 76) in gastric cancer. The overexpression of MAPK can promote proliferation and tumorigenesis in gastric cancer (77). CDK2 can regulate cell cycles (78) and aerobic glycolysis (79) in gastric cancer. Inhibition of CSNK2A1 decreased the proliferative and invasive activity of breast cancer cells (80). However, how to change the biological function by ITGB1 needs further study. Consistently, the integrin family was implicated as an important inducer of tumorigenesis, and it was significantly implicated in cancer metastasis (81), drug resistance (82), and immune evasion (83), and it was clear that ITGB1 was one of the most important integrin family members. In terms of metabolism, current studies have shown that integrin activity can regulate insulin sensitivity in adipocytes and thereby systemic metabolism (84). Meanwhile, Na KH et al. found that the hypoxia could affect integrin α4 expression to trophoblast invasion during early implantation (85). While few recent studies have focused on ITGB1 and metabolism, the specific mechanism remains to explore. We found that ITGB1 plays a crucial role in metabolic pathways and cuproptosis in DGC cells.

Devimistat (CPI-613) was one of the inhibitors of energy metabolism in mitochondria and can effectively inhibit the tricarboxylic acid cycle (86). Devimistat may affect cuproptosis by affecting the tricarboxylic acid cycle, but the specific mechanism needs further study, and it can be used as an effective targeted drug for ITGB1 related DGC. In recent study, drugs related to cuproptosis can also be used to the treatment of tumors. Disulfiram, a copper ionophore, targeted glioblastoma stem cells (87). Elesclomol can targeted treatment of melanoma (88). From this study, we found that drugs related to cuproptosis can also be used as targeted drugs for ITGB1 to treat DGC.

In summary, ITGB1 was associated with worse prognosis and regulated tumor metabolism and cuproptosis in DGC. Our findings may provide new targets for developing improved DGC therapies by influencing the cuproptosis and metabolic pathway in combination with anti-ITGB1 biotherapy.

The main limitation of this research was using the public dataset from different cohorts, which have somewhat heterogeneous in patients’ derivation and data processing. In addition, we utilized multiple genomic and transcriptomic datasets for analysis. The dataset with RNA sequencing was available in ACRG, TCGA and CPTAC cohorts and the proteomics data were obtained from PKU, and CPTAC cohorts. As a result, the association between biological process and gene expression, including analysis of metabolic reprogramming and cuproptosis pathways, needs further validation. Due to the current limited availability and difficult culture conditions of DGC cell lines, further exploration and verification were needed to perform in other GC cell lines. Moreover, drug related analysis the mutation landscape were inferred by bioinformatics methods, and the specific mechanism and dose-effect relationship were still unknown, which needs further molecular biological research and clinical trials.

## 5 Conclusion

This study discovered a new phenomenon of ITGB1 regulating cuproptosis and verified by cytological experiments. Explored in this study can enhance understanding of molecular mechanism and guide the targeted therapeutic application of ITGB1 for the DGC.

## Data availability statement

The original contributions presented in the study are included in the article/**Supplementary Material**. Further inquiries can be directed to the corresponding authors.

## Ethics statement

All studies have been approved by the Institutional Research Board.

## Author contributions

Conception and design: HC, WC, LL. Development of methodology: HC, WC. Acquisition of data (provided data, acquired and managed patients, provided facilities, etc.): HC, WC, XZ, HR, CY, MW, TM. Analysis and interpretation of data (e.g., statistical analysis, biostatistics, computational analysis): HC, WC, XX, YL. Writing, review, and/or revision of the manuscript: HC, WC, KX, JL, FD, ZZ. Administrative, technical, or material support (i.e., reporting or organizing data, constructing databases): HC, WC, LS. Study supervision: HC, LL. All authors contributed to the article and approved the submitted version.
