# Supplementary material for: ITGB1-mediated molecular landscape and cuproptosis phenotype induced the worse prognosis in diffuse gastric cancer
Source: Front Oncol. 2023 Mar 16;13:1115510. doi: 10.3389/fonc.2023.1115510 (PMC10063208; doi:10.3389/fonc.2023.1115510)
Supplement: Supplementary file 1 [file DataSheet_1.pdf]

Supplementary Material

ITGB1-mediated molecular landscape and cuproptosis phenotype induced the worse prognosis in diffuse gastric cancer

1 Supplementary Figures and Tables

1.1 Supplementary Figures

Figure S1. High and low ITGB1 was associated with intestinal GC survival.

Kaplan-Meier curves of relapse-free survival according to ITGB1 high and low groups in the ACRG intestinal GC cohort (A) and TCGA intestinal GC cohort (B).

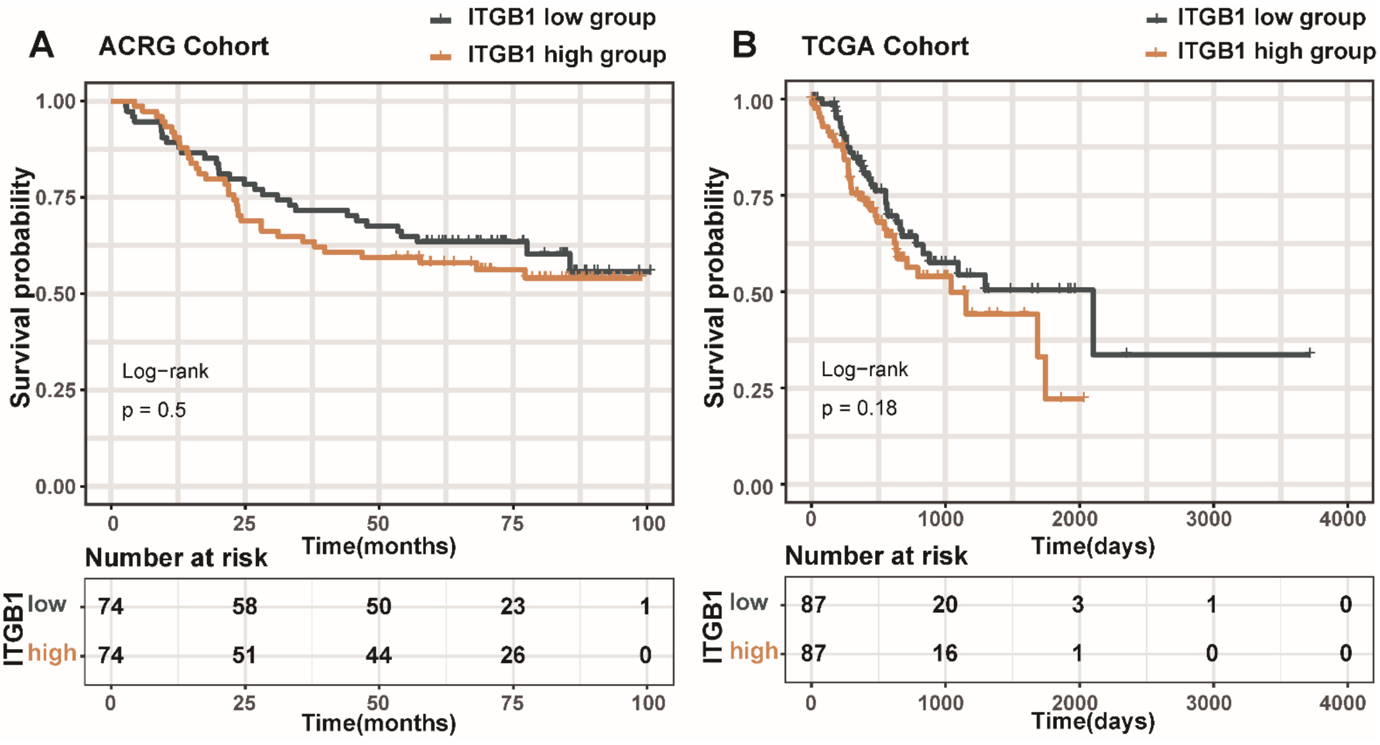

Figure S2. Tumor nonsilent mutation load and mutational signatures extracted from the DGC dataset.

(A) The tumor nonsilent mutation load in the ITGB1-low group and the ITGB1-high group. (B) The chromosomal copy number variation in the ITGB1-low and the ITGB1-high groups with oncoplot illustration. (C) The progress of automatically determines the optimal number of mutational signatures (n=4). (D) Cosine similarity analysis of extracted mutational signatures against the 30 identified signatures in Catalogue of Somatic Mutations in Cancer (COSMIC, V2) with heatmap illustration.

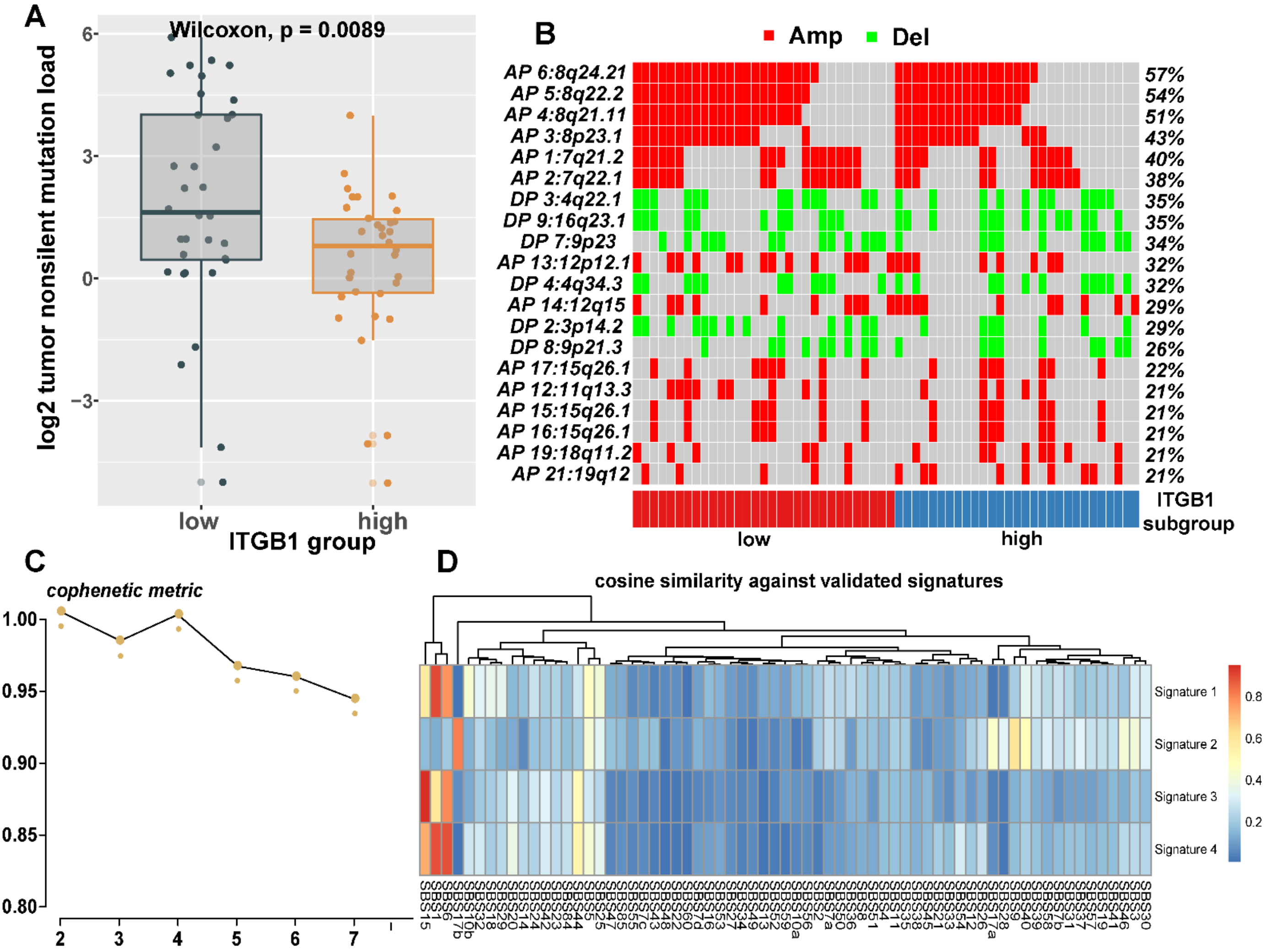

Figure S3. The landscape of biological processes of ITGB1 in diffuse GC by the joint analysis of CPTAC and PKU in the protein level.

Metascape enrichment network visualization summarized different biological processes (A), composition of the CPTAC and PKU databases (B) and the relevance (C) in the protein level. The name of biological processes showed in the right of chart. (D) The landscape of protein-protein interacting. (E) The landscaoe of protein-protein interacting between and within the MCODE clusters. (F) Heatmap of enrichment analysis of 20 biological processes associated with ITGB1 in the CPTAC and PKU. (G) Heatmap of enrichment analysis of targeted genes associated with ITGB1 in the CPTAC and PKU. (H) Heatmap of enrichment analysis of regulated genes associated with ITGB1 in the

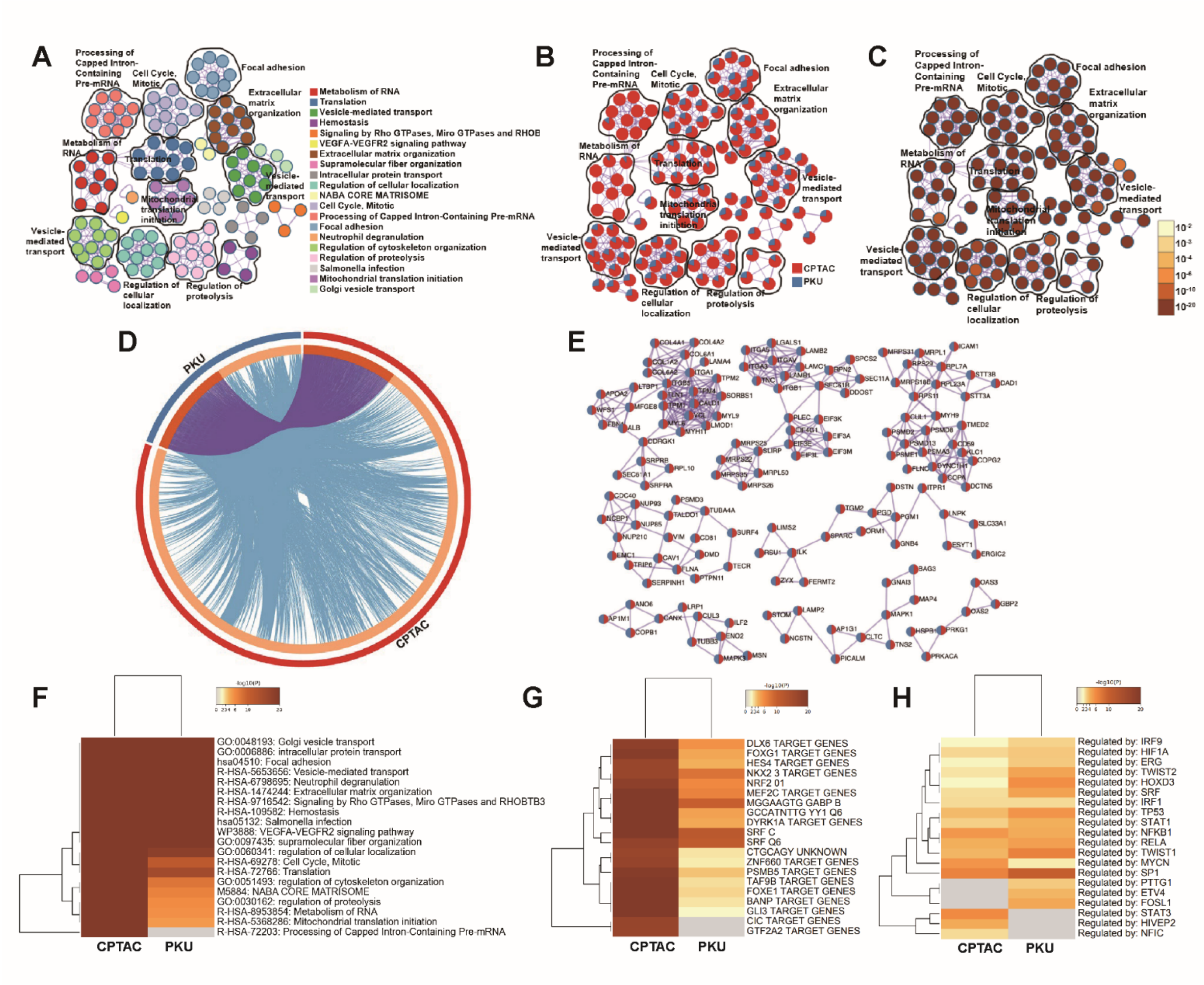

CPTAC and PKU.

Figure S4. Enrichment analysis of ITGB1 related pathways.

(A) The different clinical features and molecular subtypes in PKU cohort with ITGB1 expression subgroup. (B) The differential analysis of mRNA in ACRG and TCGA cohorts. (C) Related metabolism enriched analysis was calculated and compared in different ITGB1 groups from ACRG cohort.

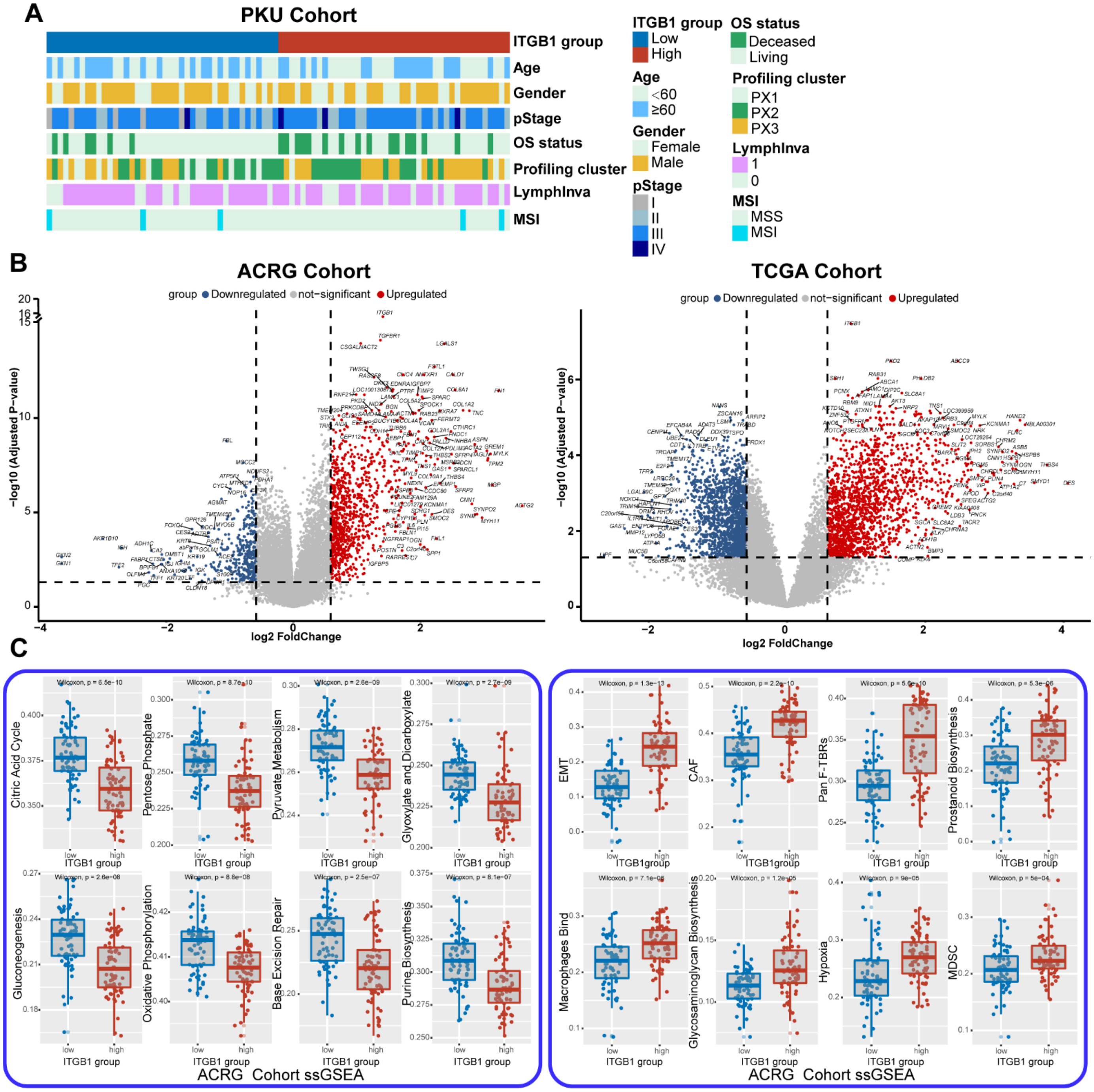

Figure S5. Kaplan-Meier curves of relapse-free survival according to cuproptosis high and low groups in TCGA cohort (A) and ACRG cohort (B). Correlation analysis between ITGB1 and FDX1 in TCGA cohort (C) and CPTAC cohort (D).

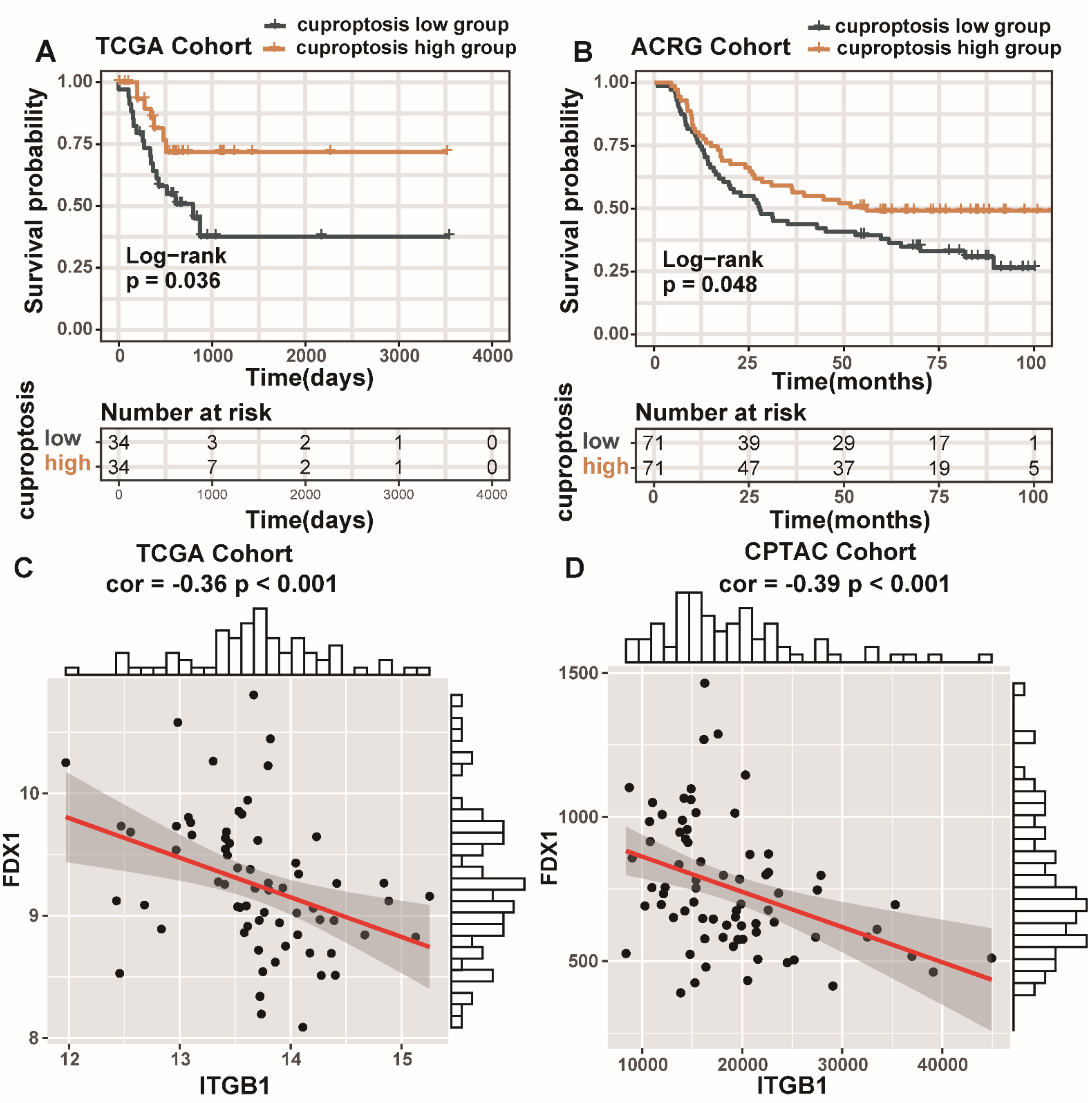

Figure S6. The CERES score and the RNAi Score evaluated the importance of ITGB1 for cell survival of the diffuse GC which was downloaded from Depmap portal. The CERES score approached to 0 meant the gene was not an essential gene for cell survival, while score approached to -1 meant the gene was an essential gene.

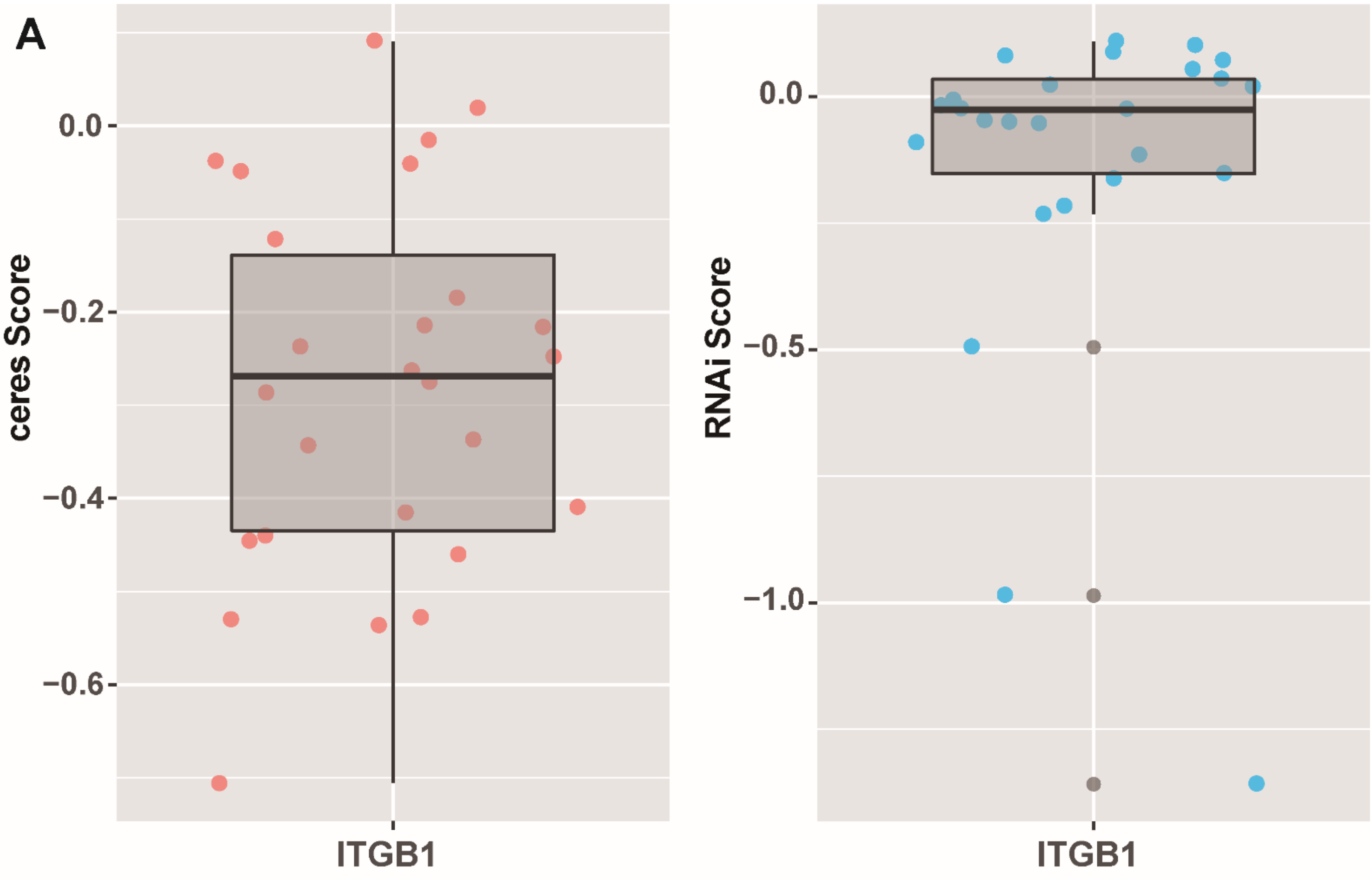

1.2 Supplementary Tables

Table S1. Clinical Characteristics of ITGB1 group in ACRG, TCGA, CPTAC and PKU cohorts.

Table S2. The phosphorylation sites associated with ITGB1 in CPTAC cohort.

Table S3. The kinases associated with ITGB1.

Table S4. The mRNA differential analysis in CPTAC, ACRG, and TCGA databases.

Table S5. Correlations between ITGB1 and the cuproptosis-related-gene (Spearman analysis).

Table S6. Correlation analysis between ITGB1 and drug sensitivity in PRISM V2 database.
